# Levels of ER Stress Markers GRP78, CHOP, and PERK in Cardiovascular Diseases

**DOI:** 10.3390/ijms27167246

**Published:** 2026-08-14

**Authors:** Tülay Oskay, Alten Oskay, Mert Özen, Isık Tekin, Fırat Okta, Abdo A. Elfiky, Murat Seyit, Atakan Yılmaz, Hakan Akça, Ibrahim Türkçüer, Gergana Lengerova, Martina Bozhkova, Steliyan Petrov, Aylin Köseler

**Affiliations:** 1 Department of Cardiology, Denizli State Hospital, 20010 Denizli, Türkiye; oskaytulay@gmail.com; 2 Department of Emergency Medicine, Faculty of Medicine, Pamukkale University, 20160 Denizli, Türkiye; aoskay@pau.edu.tr (A.O.); mert@pau.edu.tr (M.Ö.); dr.fokta@gmail.com (F.O.); mseyit@pau.edu.tr (M.S.); atakany@pau.edu.tr (A.Y.); iturkcuer@pau.edu.tr (I.T.); 3 Department of Cardiology, Faculty of Medicine, Pamukkale University, 20160 Denizli, Türkiye; itekin@pau.edu.tr; 4 Department of Biophysics, Faculty of Science, Cairo University, Giza 12613, Egypt; abdo@sci.cu.edu.eg; 5 Department of Medical Genetics, Faculty of Medicine, Pamukkale University, 20160 Denizli, Türkiye; hakca@pau.edu.tr; 6 Microbiology Laboratory, Department of Microbiology, Virology, Clinical Laboratory and Immunology, Military Medical Academy of Sofia, 1000 Sofia, Bulgaria; gergana.lengerova@yahoo.com; 7 Department of Medical Microbiology and Immunology “Prof. Dr. Elissay Yanev”, Medical University of Plovdiv, 4002 Plovdiv, Bulgaria; martina.bozhkova@mu-plovdiv.bg (M.B.); steliyan.petrov@mu-plovdiv.bg (S.P.); 8 Research Institute, Medical University of Plovdiv, 4002 Plovdiv, Bulgaria; 9 Center of Competence-Personalized Innovative Medicine, 4002 Plovdiv, Bulgaria; 10 Department of Biophysics, Faculty of Medicine, Pamukkale University, 20160 Denizli, Türkiye

**Keywords:** ER stress, GRP78, PERK, CHOP, myocardial infarction, heart failure

## Abstract

Cardiovascular diseases remain the leading cause of mortality worldwide, and endoplasmic reticulum (ER) stress has emerged as an important molecular mechanism underlying myocardial injury and heart failure. This study investigated the expression of the ER stress-related genes GRP78, PERK, and CHOP in peripheral whole blood obtained from patients with acute cardiovascular diseases. A total of 300 participants were enrolled, including 200 patients with ST-segment elevation myocardial infarction (STEMI, *n* = 55), non-ST-segment elevation myocardial infarction (NSTEMI, *n* = 88), decompensated heart failure (DHF, *n* = 40), or unstable angina pectoris (USAP, *n* = 17), and 100 healthy controls. Relative mRNA expression levels were quantified using quantitative real-time PCR. Intergroup comparisons were performed using the Kruskal–Wallis test followed by Dunn’s post hoc test with Bonferroni adjustment. Significant differences in GRP78, PERK, and CHOP expression were observed among the study groups (all *p* < 0.001). GRP78 and PERK expression levels were highest in the STEMI and DHF groups, whereas CHOP expression was highest in the STEMI group. Significant positive correlations were identified between troponin and CHOP (*r* = 0.48), GRP78 (*r* = 0.42), and PERK (*r* = 0.39) (all *p* < 0.001), while weaker but significant associations were observed between inflammatory markers (CRP and NLR) and ER stress-related gene expression. Exploratory receiver operating characteristic (ROC) analysis showed that CHOP demonstrated the highest discriminatory performance for distinguishing patients with acute cardiovascular disease from healthy controls. These findings indicate that peripheral whole-blood ER stress-related gene expression is associated with acute cardiovascular disease and correlates with established biomarkers of myocardial injury and inflammation. Further prospective studies are required to determine the clinical significance of these findings.

## 1. Introduction

Cardiovascular diseases remain a leading cause of morbidity and mortality worldwide. Increasing evidence indicates that endoplasmic reticulum (ER) stress and its associated molecular signaling pathways play important roles in the pathophysiology and progression of myocardial injury [1,2,3]. These pathologies, which are the disease groups with the highest mortality and morbidity rates worldwide, are characterized by structural deformities in the vascular wall because of dyslipidemia triggered by metabolic disorders such as genetic predisposition, obesity and insulin resistance, and an imbalance in the levels of c-peptide and adiponectin [4]. Atherosclerosis, which is especially characterized by the accumulation of lipid or fibrin plaques in the coronary arteries, is a fundamental pathological process leading to the narrowing of the lumen of the artery and to the development of acute cardiovascular events resulting from the formation of a thrombus caused by the rupture of unstable plaques [4]. Complicated lesions, such as intraplaque hemorrhage associated with atherosclerotic plaque formation, profoundly alter the expression of ER stress-related genes, thereby accelerating tissue damage and vascular remodeling [4,5]. Vascular calcification, particularly in chronic kidney disease and diabetes mellitus, contributes to the progression of arteriosclerosis through calcium deposition within the intimal and medial layers of the vascular wall [6]. Current evidence indicates that vascular calcification is not a passive degenerative process but rather a tightly regulated biological event driven by multiple interacting factors, including disturbed phosphate metabolism and oxidative stress [6]. During this process, activation of the PERK–eIF2α–ATF4–CHOP signaling pathway by tumor necrosis factor-α promotes osteogenic differentiation of vascular smooth muscle cells, thereby driving vascular mineralization [6]. In addition, metabolic disorders such as hyperhomocysteinemia induce ER stress in vascular smooth muscle cells, impair protein-folding capacity, and increase susceptibility to calcification [4]. This phenotypic transition is accompanied by reduced expression of contractile markers, including α-smooth muscle actin, and increased expression of osteogenic markers such as osteopontin and runt-related transcription factor 2 (RUNX2) [7].

The protein-folding capacity of the endoplasmic reticulum (ER), which is essential for maintaining cellular homeostasis, is disrupted by pathological conditions such as ischemia, hyperphosphatemia, oxysterol accumulation, and glycation disorders. These disturbances result in the accumulation of misfolded proteins and trigger the unfolded protein response (UPR) [8]. Initially, the UPR promotes adaptive mechanisms that restore ER homeostasis by modulating protein synthesis, enhancing the expression of molecular chaperones, and increasing the degradation of misfolded proteins. The three principal UPR signaling pathways—IRE1, PERK, and ATF6—coordinate these protective responses to alleviate proteotoxic stress [8]. However, persistent or excessive ER stress results in sustained activation of the PERK–eIF2α–ATF4 pathway, which induces transcription factors such as RUNX2, promotes the osteogenic phenotypic transition of vascular smooth muscle cells (VSMCs), and contributes to matrix maturation and pathological vascular mineralization [6,7]. In parallel, activation of CHOP, a downstream effector of the PERK pathway, promotes VSMC apoptosis by suppressing the anti-apoptotic protein BCL-2. The apoptotic bodies released from dying cells subsequently serve as nucleation sites for hydroxyapatite crystal formation, thereby accelerating vascular calcification [6].

GRP78 (also known as BiP) is a major endoplasmic reticulum (ER)-resident molecular chaperone that binds to the ER stress sensors PERK, IRE1, and ATF6 under physiological conditions, thereby maintaining them in an inactive state. During ER stress, GRP78 dissociates from these sensors and preferentially binds to misfolded proteins, initiating activation of the unfolded protein response (UPR) [9]. The release of GRP78 activates signaling pathways that transiently suppress global protein synthesis, thereby reducing the influx of newly synthesized proteins into the ER and alleviating proteotoxic stress [6,8]. In addition to its role in protein quality control, increased GRP78 expression has been associated with vascular stiffness through enhanced extracellular vesicle release and subsequent recruitment of inflammatory cells to sites of vascular calcification [6].

CHOP is a pro-apoptotic transcription factor that is markedly upregulated in advanced atherosclerotic plaques and vascular calcification. It promotes vascular smooth muscle cell apoptosis by inducing pro-apoptotic proteins such as BIM, activating calcium-dependent signaling pathways, and suppressing the expression of contractile proteins including calponin and SM22α, thereby disrupting vascular integrity [10,11]. Furthermore, CHOP activation disturbs cellular redox homeostasis, enhances reactive oxygen species production, and amplifies endothelial dysfunction and inflammatory cytokine release. In lipid-laden macrophages, CHOP also promotes intracellular calcium release and activates the caspase cascade, thereby contributing to necrotic core formation and the progression of atherosclerosis under conditions of persistent ER stress [5].

PERK is a transmembrane kinase localized to the endoplasmic reticulum (ER) membrane that serves as one of the principal sensors of ER stress. Upon activation, PERK undergoes autophosphorylation and phosphorylates eukaryotic initiation factor 2 alpha (eIF2α), resulting in a global reduction in protein synthesis and a subsequent decrease in the protein-folding burden within the ER. At the same time, selective translation of activating transcription factor 4 (ATF4) is maintained, leading to induction of CHOP, a key mediator of apoptosis implicated in pathological conditions such as vascular calcification and hypertension [12]. In addition, PERK signaling interacts with the CDK9–cyclin T1 complex to regulate ATF4 activity, thereby acting as an important molecular link in chronic kidney disease-associated vascular calcification [6]. Suppression of protein synthesis through the PERK pathway also initiates cellular reprogramming, promoting osteogenic gene expression in vascular smooth muscle cells (VSMCs) [13]. Furthermore, PERK signaling contributes to mitochondrial dysfunction by regulating calcium transfer from the ER to mitochondria and IP3R-mediated calcium leakage, leading to opening of the mitochondrial permeability transition pore and activation of apoptotic pathways [14]. Under physiological conditions, GADD34 provides negative feedback by promoting dephosphorylation of eIF2α and restoring protein synthesis. However, disruption of this regulatory mechanism promotes the transition of atherosclerotic plaques from a stable to a vulnerable phenotype and facilitates vascular stenosis [15,16].

Although accumulating evidence indicates that endoplasmic reticulum (ER) stress contributes to the pathogenesis of cardiovascular disease, the clinical significance of ER stress-related biomarkers across different acute cardiovascular conditions remains incompletely understood. Most previous studies have focused on experimental models or individual disease entities, whereas comparative clinical data encompassing acute coronary syndromes and decompensated heart failure remain limited. Therefore, the present study aimed to investigate the expression of the ER stress-related genes GRP78, PERK, and CHOP in peripheral whole blood from patients with acute cardiovascular diseases and to evaluate their associations with established biomarkers of myocardial injury and systemic inflammation.

## 2. Results

A total of 300 participants were included in the study, comprising 200 patients with cardiovascular disease and 100 healthy controls. Among the patient group, 88 (44.0%) were diagnosed with NSTEMI, 55 (27.5%) with STEMI, 40 (20.0%) with decompensated heart failure (DHF), and 17 (8.5%) with unstable angina pectoris (USAP). The demographic characteristics of the study population are presented in Table 1. The mean age was highest in the DHF group (72.75 ± 10.49 years) and lowest in the USAP group (55.00 ± 14.80 years). Male predominance was observed in the NSTEMI, STEMI, and USAP groups, whereas the sex distribution was more balanced in the DHF group. Age differed significantly among the diagnostic groups (*p* < 0.001), whereas sex distribution also showed significant intergroup variation (*p* < 0.05).

Table 2 summarizes the laboratory findings according to the diagnostic groups. Statistically significant differences were observed among the groups for WBC count, hemoglobin, neutrophil-to-lymphocyte ratio (NLR), C-reactive protein (CRP), creatinine, glucose, AST, ALT, troponin, and CK-MB levels (all *p* < 0.05), whereas platelet count, sodium, and potassium concentrations did not differ significantly. Overall group differences were assessed using the Kruskal–Wallis test, and variables showing significant overall differences were subsequently evaluated using Dunn’s post-hoc multiple comparison test with Bonferroni adjustment. Pairwise comparisons are summarized in the revised tables.

Glucose levels were elevated in all cardiovascular disease groups compared with controls, with the highest mean value observed in the STEMI group. AST and ALT levels showed marked variability and reached their highest mean values in the DHF group. Cardiac injury biomarkers also differed significantly across the diagnostic groups; troponin levels were highest in the STEMI group, whereas CK-MB levels were elevated predominantly in the NSTEMI and STEMI groups compared with the control, DHF, and USAP groups.

In contrast, platelet count (*p* = 0.612), sodium (*p* = 0.089), and potassium (*p* = 0.412) did not differ significantly among the diagnostic groups.

The mRNA expression levels of GRP78, PERK, and CHOP differed significantly among the diagnostic groups (Kruskal–Wallis test, all *p* < 0.001), as summarized in Table 3. GRP78 expression ranged from 1.00 ± 0.25 in the control group to 2.50 ± 0.80 in the STEMI group. Intermediate expression levels were observed in the NSTEMI (1.80 ± 0.60) and DHF (2.30 ± 0.70) groups, whereas the USAP group exhibited expression levels comparable to those of the control group. PERK expression showed a similar distribution, ranging from 1.00 ± 0.20 in controls to 2.20 ± 0.70 in STEMI patients, with intermediate expression levels in the NSTEMI (1.60 ± 0.50) and DHF (2.00 ± 0.60) groups. CHOP expression also differed significantly among the study groups, ranging from 1.00 ± 0.20 in controls to 3.00 ± 1.00 in STEMI patients, while the NSTEMI (1.90 ± 0.70) and DHF (1.70 ± 0.60) groups demonstrated intermediate expression levels.

**Figure 1:**
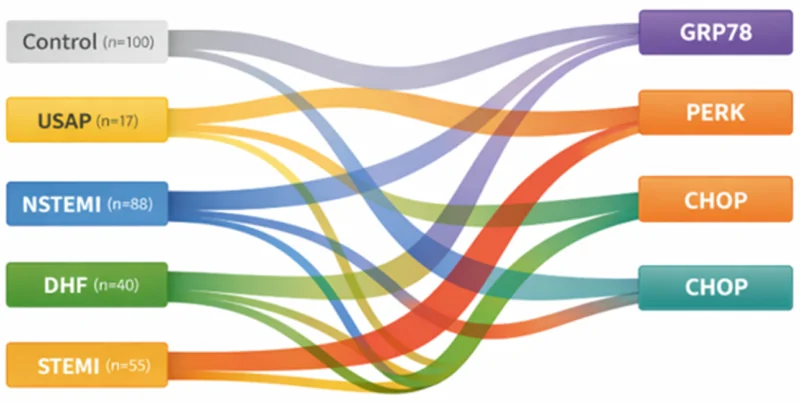
Sankey diagram demonstrating the graded increase in ER stress-related gene (GRP78, PERK, and CHOP) expression from healthy controls to severe cardiovascular conditions. Flow thickness represents the relative expression burden (sample size × mean expression). Minimal activation is observed in the control and USAP groups, whereas a marked increase is evident in the NSTEMI and DHF groups, with peak expression in the STEMI group, highlighting the association between ER stress activation and disease severity.

To visually summarize the distribution of ER stress-related gene expression across diagnostic categories, a Sankey diagram was constructed (Figure 1). The diagram integrates sample size and mean mRNA expression values by scaling link widths as n × mean expression, thereby reflecting the relative group-level ER stress burden. As illustrated, expression of GRP78, PERK, and particularly CHOP was markedly elevated in the STEMI group compared with other diagnostic categories. The NSTEMI and DHF groups demonstrated intermediate activation, whereas the USAP group exhibited consistently lower expression levels across all three markers. The visualization highlights the pronounced CHOP upregulation in STEMI patients and supports the graded pattern of ER stress activation observed across the clinical spectrum.

Spearman correlation analysis was performed to evaluate the associations between cardiac injury biomarkers, inflammatory markers, and peripheral whole-blood ER stress-related gene expression levels. Troponin levels showed significant positive correlations with CHOP (*r* = 0.48, *p* < 0.001), GRP78 (*r* = 0.42, *p* < 0.001), and PERK expression (*r* = 0.39, *p* < 0.001), indicating that greater biochemical evidence of myocardial injury was associated with higher peripheral whole-blood expression of ER stress-related genes. Similarly, CK-MB levels were positively correlated with CHOP (*r* = 0.36, *p* = 0.002), GRP78 (*r* = 0.31, *p* = 0.006), and PERK expression (*r* = 0.29, *p* = 0.011).

Among the inflammatory markers, CRP demonstrated weak but statistically significant positive correlations with GRP78 (*r* = 0.28, *p* = 0.010) and CHOP expression (*r* = 0.21, *p* = 0.048), whereas its association with PERK did not reach statistical significance (*r* = 0.19, *p* = 0.061). In addition, NLR showed significant positive correlations with CHOP (*r* = 0.33, *p* = 0.004), GRP78 (*r* = 0.27, *p* = 0.012), and PERK expression (*r* = 0.25, *p* = 0.018).

Overall, these findings demonstrate significant associations between conventional biomarkers of myocardial injury and inflammation and peripheral whole-blood ER stress-related gene expression, with CHOP showing the strongest correlations among the evaluated markers. The correlation results are summarized in Table 4.

Spearman correlation analysis was used to evaluate associations between variables. Correlation strength was interpreted as weak (r < 0.3), moderate (0.3–0.5), and strong (r > 0.5). A *p*-value < 0.05 was considered statistically significant.

**Figure 2:**
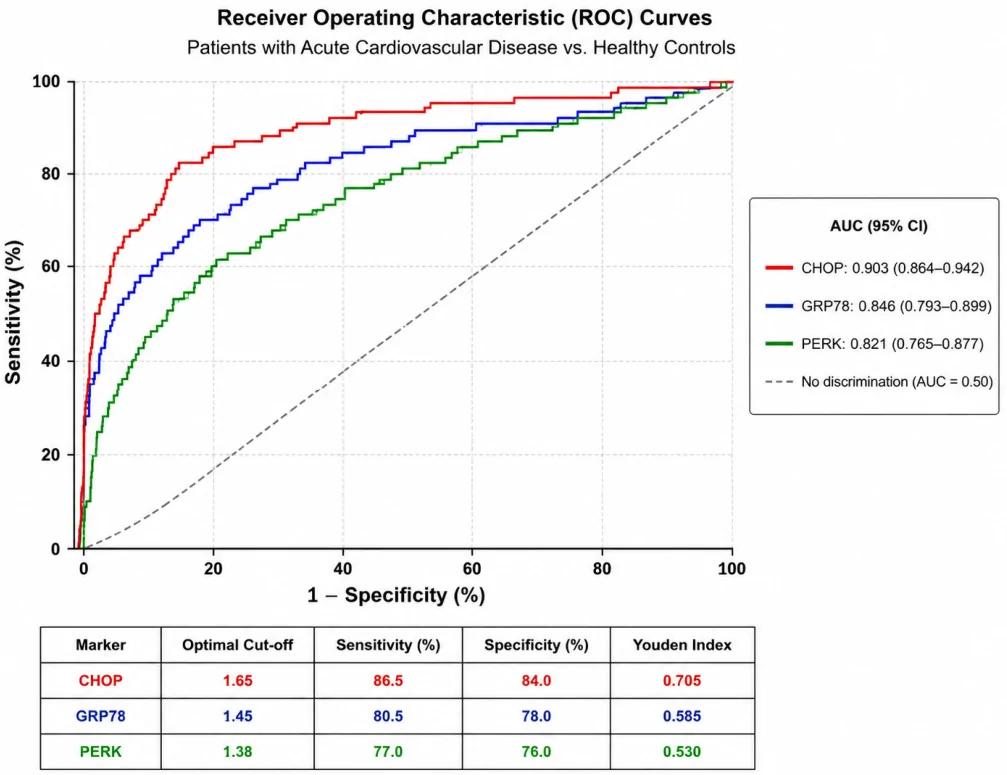
Receiver operating characteristic (ROC) curves showing the exploratory discriminatory performance of peripheral whole-blood GRP78, PERK, and CHOP mRNA expression levels in distinguishing patients with acute cardiovascular disease from healthy controls. The diagonal dashed line represents the line of no discrimination (AUC = 0.50). AUC, area under the curve; CI, confidence interval.

Receiver operating characteristic (ROC) curve analysis was performed as an exploratory analysis to evaluate the discriminatory performance of peripheral whole-blood GRP78, PERK, and CHOP mRNA expression levels in distinguishing patients with acute cardiovascular disease from healthy controls. Among the three markers, CHOP demonstrated the highest discriminatory performance, followed by GRP78 and PERK. The area under the ROC curve (AUC), 95% confidence intervals (95% CIs), optimal cut-off values, sensitivity, and specificity are summarized in Table 5, whereas the ROC curves are presented in Figure 2.

## 3. Discussion

The present study demonstrates significant differences in peripheral whole-blood expression of the ER stress-related genes GRP78, PERK, and CHOP among patients with acute cardiovascular diseases. These findings support the involvement of systemic ER stress responses in the pathophysiology of acute cardiovascular conditions and are consistent with the growing body of evidence implicating ER stress in myocardial injury and heart failure. The particularly pronounced CHOP expression observed in STEMI patients may reflect enhanced activation of apoptosis-related ER stress signaling during acute myocardial injury. However, given the cross-sectional design of the present study, these findings should be interpreted as associations rather than evidence of causal mechanisms.

Our present findings are also consistent with our previous study investigating the clinical significance of ER stress markers in patients with heart failure with reduced ejection fraction (HFrEF) [3]. In that study, GRP78, PERK, and particularly CHOP expression levels were significantly associated with disease severity, duration of hospital stay, and the risk of decompensation [3]. Similarly, the elevated expression levels of GRP78 (2.30 ± 0.70) and PERK (2.00 ± 0.60) observed in the decompensated heart failure group in the present study further support the involvement of systemic ER stress responses in the pathophysiology of heart failure decompensation.

The significantly elevated expression of GRP78, PERK, and particularly CHOP in peripheral whole blood from STEMI patients suggests enhanced systemic activation of ER stress-related pathways during acute myocardial ischemia. Among these markers, CHOP demonstrated the greatest increase and showed the strongest correlation with troponin levels, suggesting a close association between ER stress-mediated apoptosis and the extent of myocardial injury. These findings are consistent with previous studies demonstrating activation of the PERK/eIF2α/ATF4/CHOP pathway during ischemic stress, suggesting that severe ischemia promotes a shift from adaptive unfolded protein response signaling toward apoptosis [6,10,11,12].

Interestingly, although GRP78 and PERK expression levels were higher in the DHF group than in the NSTEMI group, CHOP expression was higher in NSTEMI patients than in DHF patients. This difference may reflect the distinct biological role of CHOP as a downstream pro-apoptotic transcription factor within the unfolded protein response. Whereas GRP78 functions primarily as an ER chaperone that promotes adaptive protein folding and PERK mediates the early stress response, CHOP is preferentially induced during prolonged or severe ER stress and drives apoptotic signaling through the PERK/eIF2α/ATF4 pathway [5,6,10,11,12]. Therefore, the relatively higher CHOP expression observed in NSTEMI patients may indicate greater activation of apoptosis-related pathways during acute myocardial injury, whereas decompensated heart failure may predominantly exhibit persistent adaptive ER stress responses without a proportional increase in CHOP expression [1,6,11]. Nevertheless, these mechanistic interpretations remain speculative and should be confirmed in future mechanistic and protein-level studies.

Troponin and CK-MB levels showed significant positive correlations with all three ER stress markers, indicating that higher peripheral whole-blood expression of GRP78, PERK, and CHOP was associated with greater biochemical evidence of myocardial injury. Furthermore, CRP and NLR were positively associated with GRP78, PERK, and CHOP expression, supporting a close interplay between systemic inflammation and ER stress-related pathways. Collectively, these findings suggest that systemic ER stress, as reflected by peripheral whole-blood mRNA expression, may represent an important molecular interface linking myocardial injury and inflammatory responses in acute cardiovascular conditions [1,4,5,6].

The significant associations observed between CRP and both GRP78 and CHOP expression are consistent with previous studies indicating that ER stress is closely interconnected with systemic inflammatory processes in cardiovascular disease [4,17,18]. The elevated expression of GRP78 and PERK observed in the decompensated heart failure group is consistent with previous studies suggesting persistent activation of systemic ER stress responses and dysregulation of the unfolded protein response (UPR) in chronic heart failure [19]. The relatively advanced age of the DHF group in the present study (72.75 years) may also have contributed to these findings, as aging has been associated with progressive impairment of proteostasis and altered regulation of key UPR components, including PERK [1]. Therefore, the observed expression profile may reflect the combined effects of chronic heart failure and age-related alterations in ER stress signaling.

The relatively low expression levels observed in the USAP group, which were comparable to those of healthy controls, are consistent with the less extensive myocardial injury typically observed in unstable angina compared with myocardial infarction. The association between CRP and GRP78 further supports the close relationship between systemic inflammation and ER stress-related pathways reported in previous studies [4,18]. Although exploratory ROC analysis suggested that these markers may possess discriminatory potential, the present cross-sectional study was not designed to establish diagnostic performance or clinical utility. Consequently, these findings should be considered hypothesis-generating and require confirmation in prospective studies with independent validation cohorts.

Endoplasmic reticulum stress is closely linked to mitochondrial function through its structural and functional interaction with mitochondria, and disruption of this crosstalk has been implicated in myocardial ischemia and heart failure [14,20]. Previous experimental studies have demonstrated that impaired Ca^2+^ transfer between the ER and mitochondria contributes to mitochondrial dysfunction, oxidative stress, and apoptosis during ischemic injury [14,20]. The elevated peripheral whole-blood expression of GRP78 and PERK observed in the present study is consistent with activation of systemic ER stress responses in patients with acute cardiovascular disease. However, because gene expression was measured in peripheral blood rather than myocardial tissue, these findings should not be interpreted as direct evidence of myocardial mitochondrial dysfunction or tissue-specific ER stress activation [21,22].

Exploratory ROC analysis further demonstrated that CHOP exhibited the highest discriminatory performance for distinguishing patients with acute cardiovascular disease from healthy controls, followed by GRP78 and PERK (Table 5; Figure 2). Although these findings suggest that peripheral whole-blood ER stress-related gene expression may have discriminatory potential, the present study was not designed as a diagnostic accuracy study. Therefore, the observed ROC performance should be interpreted cautiously and requires validation in independent prospective cohorts before clinical application.

The current medical treatment of the study participants may also have influenced basal ER stress-related gene expression. Previous studies have shown that beta-blockers and statins can attenuate ER stress signaling by modulating the GRP78/PERK/CHOP pathway [11,16]. Consequently, differences in medication use may have contributed to the relatively lower expression levels observed in the USAP group. Furthermore, as we recently demonstrated in a pharmacogenomic study of a similar emergency cardiac cohort, substantial interindividual variability in CYP450 metabolizer phenotypes, particularly involving CYP2D6 and CYP3A4, may influence drug exposure and consequently modulate systemic ER stress responses [23].

Finally, the higher mean age of patients in the decompensated heart failure group (72.75 years) should be considered when interpreting the results. Aging has been associated with impaired proteostasis and altered regulation of unfolded protein response pathway components, including GRP78 and PERK, which may contribute to sustained systemic ER stress responses in elderly patients with heart failure [1].

The observed alterations in systemic ER stress-related gene expression provide further support for investigating ER stress pathways as potential therapeutic targets in cardiovascular disease. Experimental studies have shown that chemical chaperones, including 4-phenylbutyric acid (4-PBA), and several natural compounds can attenuate ER stress signaling and reduce myocardial injury by modulating the PERK–ATF4–CHOP pathway [1,11,24]. Although the elevated CHOP expression observed in the present study is consistent with these experimental findings, the current cross-sectional design does not permit conclusions regarding therapeutic responsiveness. Therefore, future mechanistic and interventional studies are required to determine whether modulation of ER stress pathways can improve clinical outcomes in patients with acute cardiovascular disease.

Overall, our findings demonstrate significant associations between peripheral whole-blood GRP78, PERK, and CHOP mRNA expression and established biomarkers of myocardial injury and inflammation. Exploratory ROC analysis further indicated that CHOP exhibited the highest discriminatory performance among the evaluated markers, followed by GRP78 and PERK. Nevertheless, these findings should be interpreted cautiously because the present study was not designed as a diagnostic accuracy study, and the ROC analysis was exploratory. Validation in independent prospective cohorts is required before these markers can be considered for clinical application.

In conclusion, the present study demonstrates that systemic ER stress-related gene expression differs significantly among patients with acute cardiovascular diseases and is associated with conventional biomarkers of myocardial injury and inflammation. These findings support the involvement of systemic ER stress responses in the pathophysiology of acute cardiovascular disease. However, further longitudinal, mechanistic, and protein-based studies are required to determine the diagnostic, prognostic, and therapeutic relevance of these findings.

### Limitations

This study has several limitations that should be acknowledged. First, the cross-sectional design limits the ability to establish causal relationships between systemic ER stress responses and acute cardiovascular disease and precludes conclusions regarding prognostic significance or risk stratification. Second, protein-level validation (e.g., ELISA or Western blot) was not performed, and the findings were based solely on mRNA expression. Therefore, the observed transcriptional changes cannot be assumed to directly reflect protein abundance or biological activity. Third, although a healthy control group was included, the absence of longitudinal follow-up data precluded the evaluation of temporal changes and the prognostic significance of ER stress-related gene expression. Fourth, the interval between symptom onset and blood sample collection was not systematically recorded. Because circulating troponin and CK-MB concentrations vary substantially according to the timing of presentation, this factor may have influenced both the comparisons among diagnostic groups and the observed correlations with ER stress-related gene expression. Fifth, the present study focused on acute cardiovascular conditions and did not include patients with stable atherosclerotic disease. In addition, lipid profile parameters were not systematically evaluated; therefore, the relationship between systemic ER stress-related gene expression and the extent of atherosclerotic burden could not be assessed. Additionally, potential confounding effects of medications such as beta-blockers and statins on ER stress pathways could not be fully controlled. Future studies should ideally integrate pharmacogenomic profiling, given our recent findings that significant proportions of Turkish emergency cardiac patients exhibit non-normal metabolizer phenotypes (e.g., 55.6% for CYP2D6 and 30.4% for CYP3A4), which could substantially alter individual drug exposure and subsequent ER stress modulation [23]. Finally, ER stress-related gene expression was measured in peripheral whole blood rather than myocardial tissue. Therefore, the observed transcript levels should be interpreted as indicators of systemic circulating ER stress responses and cannot be considered direct measures of myocardial ER stress. Future prospective studies incorporating protein-level validation, comprehensive lipid profiling, patients representing different stages of atherosclerotic cardiovascular disease, and longitudinal follow-up are warranted to further establish the biological and clinical significance of these findings.

## 4. Materials and Methods

### 4.1. Study Design

This study was designed as a single-center cross-sectional observational study. The study was conducted in the Emergency Department, School of Medicine, Pamukkale University. Molecular analyses were carried out in the laboratory of the Department of Biophysics, School of Medicine, Pamukkale University. The study was conducted in accordance with the principles of the Helsinki Declaration after obtaining approval from the Non-Interventional Clinical Research Ethics Committee of Pamukkale University (No. E-60116787-020-759128; date: 30 September 2025).

### 4.2. Study Population

A total of 300 participants aged 18–80 years were included in this cross-sectional study. Of these, 200 consecutive patients admitted to the emergency department and diagnosed with cardiovascular disease were assigned to the NSTEMI, STEMI, decompensated heart failure (DHF), or unstable angina pectoris (USAP) groups according to their final clinical diagnosis. In addition, 100 apparently healthy volunteers without known cardiovascular disease were included as the control group.

Healthy controls were recruited from individuals with no clinical evidence or history of acute or chronic cardiovascular disease, active infection, malignancy, autoimmune disease, organ failure, or pregnancy. Control participants were frequency-matched to the patient population as closely as possible in terms of age and sex distribution. Written informed consent was obtained from all participants or their legal representatives. The control group consisted of 100 healthy volunteers with no clinical evidence or prior history of coronary artery disease, heart failure, acute inflammatory disease, malignancy, autoimmune disorder, severe renal or hepatic dysfunction, or ongoing infectious disease. Individuals receiving intensive cardiovascular treatment or presenting with abnormal acute-phase clinical findings were not included in the control group.

### 4.3. Clinical Data

Clinical data were recorded through a standardized Case Report Form (CRF). In this scope, patients’ sociodemographic characteristics (age and sex), medical history (hypertension, diabetes mellitus, coronary artery disease, atrial fibrillation, smoking, and alcohol use), vital signs at the time of admission, laboratory parameters, imaging results, emergency diagnoses, treatments applied, and discharge, hospitalization, and mortality information were recorded. No interventions influencing the decision of the primary physician during the diagnosis and treatment process were performed, and the study was conducted purely on an observational basis.

### 4.4. Collection of Blood Samples and Laboratory Analysis

While routine blood tests were being done at the time of an emergency service application, an additional venous blood sample was drawn into a hemogram tube for mRNA level analysis. Additional invasive procedures were not performed on the patients within the scope of the study. Samples were stored at −80 °C until analysis. Peripheral venous blood samples were collected from both patient and healthy control groups. Total RNA isolation, cDNA synthesis, and quantitative real-time PCR analyses for GRP78, PERK, and CHOP expression were performed using the same protocol for all study participants. High-sensitivity cardiac troponin I (hs-cTnI) was measured using the ARCHITECT STAT High-Sensitivity Troponin-I assay on an ARCHITECT i2000SR immunoassay analyzer (Abbott Laboratories, Abbott Park, IL, USA), and results were reported in ng/L.

### 4.5. RNA Isolation, cDNA Synthesis, and Quantitative Real-Time PCR Analysis

Total RNA isolation from whole blood samples was performed using the Qiagen RNeasy^®^ Mini Kit (Qiagen, Hilden, Germany) according to the manufacturer’s instructions. Concentrations and purity levels of the isolated RNA extracts were determined using a NanoDrop 2000 spectrophotometer (Thermo Scientific, Waltham, MA, USA) and samples that were at the appropriate purity ratio were stored at −80 °C until the analysis. Complementary DNA (cDNA) synthesis from total RNA samples was performed using the High-Capacity cDNA Reverse Transcription Kit (Applied Biosystems, Foster City, CA, USA) following the manufacturer’s protocol.

The mRNA expression levels of the genes GRP78, PERK and CHOP were determined using the quantitative Real-Time PCR (qPCR) method and PowerUp™ SYBR^®^ Green Master Mix (Applied Biosystems, USA). The GAPDH gene was used as an internal control (housekeeping gene). The obtained Ct values were normalized to GAPDH, and relative gene expression levels were calculated using the 2^−ΔΔCt^ method.

### 4.6. Statistical Analysis

Statistical analyses were performed using IBM SPSS Statistics version 24.0 (IBM Corp., Armonk, NY, USA). Continuous variables were expressed as mean ± standard deviation (SD), whereas categorical variables were presented as frequencies and percentages. Normality was assessed using the Shapiro–Wilk test and visual inspection of histograms and Q–Q plots. Because several variables demonstrated non-normal distributions and considerable variability, intergroup comparisons were performed using the Kruskal–Wallis test. When a significant overall difference was identified, Dunn’s post-hoc multiple comparison test with Bonferroni adjustment was applied for pairwise comparisons. Categorical variables were compared using the chi-square test, and associations between laboratory parameters and ER stress-related gene expression levels were evaluated using Spearman’s rank correlation analysis.

Receiver operating characteristic (ROC) curve analysis was performed as an exploratory analysis to evaluate the discriminatory performance of peripheral whole-blood GRP78, PERK, and CHOP mRNA expression levels for distinguishing patients with acute cardiovascular disease from healthy controls. The area under the ROC curve (AUC) and its 95% confidence interval (95% CI) were calculated for each marker. The optimal cut-off value was determined using the Youden index, and the corresponding sensitivity and specificity were calculated.

A two-sided *p* value < 0.05 was considered statistically significant.

## 5. Conclusions

In conclusion, peripheral whole-blood expression of GRP78, PERK, and CHOP differed significantly among patients with acute cardiovascular diseases and was associated with established biomarkers of myocardial injury and inflammation. These findings support the involvement of systemic ER stress responses in acute cardiovascular disease. Further longitudinal, mechanistic, and protein-level studies are required to validate these observations and determine their clinical significance.

## Figures and Tables

**Table 1:** Demographic characteristics of the study population, including the healthy control group.

Diagnosis	N (300)	Mean Age ± SD (Years)	Male n (%)	Female n (%)
**Control**	100	60.20 ± 12.40	58 (58.0%)	42 (42.0%)
**NSTEMI**	88	66.30 ± 13.85	56 (63.6%)	32 (36.4%)
**STEMI**	55	63.67 ± 13.88	43 (78.2%)	12 (21.8%)
**DHF**	40	72.75 ± 10.49	19 (47.5%)	21 (52.5%)
**USAP**	17	55.00 ± 14.80	14 (82.4%)	3 (17.6%)

**Table 2:** Laboratory parameters of the study population including healthy controls (mean ± SD).

Parameter	Control (n = 100)	NSTEMI (n = 88)	STEMI (n = 55)	DHF (n = 40)	USAP (n = 17)	*p*-Value
**WBC (×10^9^/L)**	7.20 ± 1.80	9.88 ± 4.07	10.82 ± 3.91	11.14 ± 7.40	8.92 ± 2.27	<0.001
**Hemoglobin (g/dL)**	14.10 ± 1.60	13.25 ± 2.39	13.97 ± 2.33	11.52 ± 2.02	14.35 ± 1.72	<0.001
**Platelet count (×10^9^/L)**	245.00 ± 60.00	237.43 ± 74.85	255.51 ± 109.93	260.80 ± 141.91	263.35 ± 58.75	0.612
**NLR (ratio)**	2.10 ± 0.90	5.33 ± 5.73	4.69 ± 3.30	8.28 ± 6.35	2.98 ± 1.17	<0.001
**CRP (mg/L)**	3.20 ± 2.10	19.08 ± 28.32	25.37 ± 51.69	38.74 ± 59.18	4.29 ± 5.83	<0.001
**Creatinine (mg/dL)**	0.90 ± 0.20	1.31 ± 1.20	0.99 ± 0.26	1.72 ± 1.24	1.05 ± 0.21	<0.001
**Glucose (mg/dL)**	95.00 ± 12.00	149.10 ± 75.06	173.84 ± 96.86	158.90 ± 86.26	125.41 ± 60.01	<0.001
**AST (U/L)**	22.00 ± 8.00	36.76 ± 28.79	43.71 ± 52.91	198.00 ± 646.77	26.06 ± 11.38	0.012
**ALT (U/L)**	20.00 ± 7.00	23.18 ± 17.00	27.00 ± 31.78	114.45 ± 342.01	22.88 ± 13.27	0.021
**Sodium (mmol/L)**	139.00 ± 2.50	137.66 ± 4.11	138.27 ± 3.39	136.50 ± 4.32	138.24 ± 2.28	0.089
**Potassium (mmol/L)**	4.30 ± 0.30	4.54 ± 0.52	4.51 ± 0.72	4.54 ± 0.54	4.54 ± 0.62	0.412
**hs-cTnI (ng/L)**	5.00 ± 2.00	287.72 ± 503.16	333.38 ± 881.52	70.50 ± 95.52	10.43 ± 6.71	<0.001
**CK-MB (ng/mL)**	1.50 ± 0.50	16.13 ± 30.35	14.87 ± 38.37	3.96 ± 3.81	1.76 ± 0.62	<0.001

WBC, white blood cell count; NLR, neutrophil-to-lymphocyte ratio; CRP, C-reactive protein; AST, aspartate aminotransferase; ALT, alanine aminotransferase; hs-cTnI, high-sensitivity cardiac troponin I; CK-MB, creatine kinase-MB. Data are presented as mean ± SD.

**Table 3:** mRNA Expression of ER Stress-Related Genes According to Diagnostic Group.

Gene	Control (n = 100)	NSTEMI (n = 88)	STEMI (n = 55)	DHF (n = 40)	USAP (n = 17)	Overall *p* Value	Post-Hoc Comparison
**GRP78**	1.00 ± 0.25	1.80 ± 0.60	2.50 ± 0.80	2.30 ± 0.70	1.00 ± 0.30	<0.001	C vs. N < 0.001; C vs. S < 0.001; C vs. D < 0.001; U vs. N < 0.001; U vs. S < 0.001; U vs. D < 0.001; N vs. S = 0.012; N vs. D = 0.041; S vs. D = 0.286
**PERK**	1.00 ± 0.20	1.60 ± 0.50	2.20 ± 0.70	2.00 ± 0.60	1.00 ± 0.25	<0.001	C vs. N = 0.004; C vs. S < 0.001; C vs. D < 0.001; U vs. N = 0.006; U vs. S < 0.001; U vs. D < 0.001; N vs. S = 0.018; N vs. D = 0.031; S vs. D = 0.354
**CHOP**	1.00 ± 0.20	1.90 ± 0.70	3.00 ± 1.00	1.70 ± 0.60	1.00 ± 0.30	<0.001	C vs. N = 0.002; C vs. S < 0.001; C vs. D = 0.009; U vs. N = 0.003; U vs. S < 0.001; U vs. D = 0.011; N vs. S = 0.008; D vs. S = 0.004; N vs. D = 0.437

**C**, healthy control; **N**, non-ST-segment elevation myocardial infarction (NSTEMI); **S**, ST-segment elevation myocardial infarction (STEMI); **D**, decompensated heart failure (DHF); **U**, unstable angina pectoris (USAP); **SD**, standard deviation. Data are presented as mean ± SD. Overall group differences were assessed using the Kruskal–Wallis test. Pairwise comparisons were performed using Dunn’s post-hoc test with Bonferroni adjustment. Adjusted *p* values are reported for statistically significant pairwise comparisons.

**Table 4:** Correlation analysis between cardiac injury markers, inflammatory parameters, and ER stress-related gene expression levels.

Variable Pair	Correlation Coefficient (r)	*p*-Value
**Troponin–CHOP**	0.48	<0.001
**Troponin–GRP78**	0.42	<0.001
**Troponin–PERK**	0.39	<0.001
**CK-MB–CHOP**	0.36	0.002
**CK-MB–GRP78**	0.31	0.006
**CK-MB–PERK**	0.29	0.011
**CRP–GRP78**	0.28	0.010
**CRP–CHOP**	0.21	0.048
**CRP–PERK**	0.19	0.061
**NLR–CHOP**	0.33	0.004
**NLR–GRP78**	0.27	0.012
**NLR–PERK**	0.25	0.018

**Table 5:** Receiver Operating Characteristic (ROC) Analysis of GRP78, PERK, and CHOP Expression in Distinguishing Patients with Acute Cardiovascular Disease from Healthy Controls.

Marker	AUC (95% CI)	Optimal Cut-Off	Sensitivity (%)	Specificity (%)	*p* Value
**GRP78**	**0.846 (0.793–0.899)**	1.45	80.5	78.0	<0.001
**PERK**	**0.821 (0.765–0.877)**	1.38	77.0	76.0	<0.001
**CHOP**	**0.903 (0.864–0.942)**	1.65	86.5	84.0	<0.001

## Data Availability

The original contributions presented in this study are included in the article. Further inquiries can be directed to the corresponding author.

## Abbreviations

The following abbreviations are used in this manuscript:

ALT	Alanine Aminotransferase
ANOVA	Analysis of Variance
AST	Aspartate Aminotransferase
ATF4	Activating Transcription Factor 4
ATF6	Activating Transcription Factor 6
BCL-2	B-cell Lymphoma 2
BiP	Binding Immunoglobulin Protein
Ca^2+^	Calcium Ion
CDK9	Cyclin-Dependent Kinase 9
cDNA	Complementary DNA
CK-MB	Creatine Kinase–Myocardial Band
CRF	Case Report Form
CRP	C-Reactive Protein
Ct	Cycle Threshold
DHF	Decompensated Heart Failure
eIF2α	Eukaryotic Translation Initiation Factor 2 Alpha
ER	Endoplasmic Reticulum
GADD34	Growth Arrest and DNA Damage-Inducible Protein 34
GAPDH	Glyceraldehyde 3-Phosphate Dehydrogenase
GRP78	Glucose-Regulated Protein 78
HF	Heart Failure
HFrEF	Heart Failure with Reduced Ejection Fraction
IP3R	Inositol 1,4,5-Trisphosphate Receptor
IRE1	Inositol-Requiring Enzyme 1
MI	Myocardial Infarction
mRNA	Messenger RNA
NanoDrop	NanoDrop Spectrophotometer
NLR	Neutrophil-to-Lymphocyte Ratio
NSTEMI	Non-ST-Segment Elevation Myocardial Infarction
PERK	Protein Kinase RNA-like Endoplasmic Reticulum Kinase
qPCR	Quantitative Real-Time Polymerase Chain Reaction
ROC	Receiver Operating Characteristic
Runx2	Runt-Related Transcription Factor 2
SD	Standard Deviation
SPSS	Statistical Package for the Social Sciences
STEMI	ST-Segment Elevation Myocardial Infarction
TNF-α	Tumor Necrosis Factor Alpha
UPR	Unfolded Protein Response
USAP	Unstable Angina Pectoris
VSMC	Vascular Smooth Muscle Cell
WBC	White Blood Cell
